# Postpartum depression and associated factors among mothers who gave birth in the last twelve months in Ankesha district, Awi zone, North West Ethiopia

**DOI:** 10.1186/s12884-019-2594-y

**Published:** 2019-11-21

**Authors:** Solomon Shitu, Biftu Geda, Merga Dheresa

**Affiliations:** 1 Wolkite University College of Health and Medical Sciences, Wolkite, Ethiopia; 2 College of Health and Medical Sciences, School of Nursing and Midwifery, Haramaya University, Harar, Ethiopia

**Keywords:** Postpartum depression, Postpartum mother, Ankesha

## Abstract

**Background:**

Postpartum depression is the most common complication of childbearing age women and is a considerable public health problem. The transition into motherhood is a difficult period that involves significant changes in the psychological, social and physiological aspects, and has increased vulnerability for the development of mental illness. More than 1 in 10 pregnant women and 1 in 20 postnatal women in Ethiopia suffer from undetected depression.

**Methods:**

Community based cross sectional study was conducted among 596 postpartum mothers in Ankesha District, North West Ethiopia, from February 01 to March 2, 2018. One stage cluster sampling technique was employed to get the study participants. The objective was to assess the prevalence and associated factors of postpartum depression among mothers who gave birth in the last Twelve months in Ankesha District, Awi Zone, North West Ethiopia, 2018. The interviewer-administered questionnaire was used to collect data and Eden Burg Postpartum Depression Scale was used to assess postpartum depression with cutoff point ≥8. The data were entered into Epi data version 3.1 and exported to SPSS version 24 for analysis. All variables with *P* < 0.25 in the bivariate analysis were included in the final model and statistical significance was declared at *P* < 0.05.

**Result:**

In this study, a total of 596 study participants were involved making a response rate of 97.4%, the prevalence of postpartum depression was 23.7% with 95%CI: 20.3–27.2. From the participant mothers who are divorced/widowed/unmarried (AOR = 3.45 95%CI: 1.35–8.82), unwanted pregnancy (AOR = 1.95 95%CI: 1.14–3.33), unpreferred infant sex (AOR = 1.79 95%CI: 1.13–2.86), infant illness (AOR = 2.08 95%CI: 1.30–3.34) and low social support (AOR = 3.16 95% CI: 1.55–6.43) was independent predictors of postpartum depression.

**Conclusion:**

Almost a quarter (23.7%) of women suffers from postpartum depression. Marital status, unwanted pregnancy, unwanted infant sex, infant illness, and low social support were independent predictors of postpartum depression. Therefore, integration of mental illness with maternal and child health care is important, information communication education and behavioral change communications on postpartum depression are better been given attention.

## Background

Postpartum depression (PPD) is a term applied to describe depressive symptoms occurring during the first year of the postpartum period and is characterized by low mood, loss of enjoyment, reduced energy, and activity, marked functional impairment, reduced self-esteem, ideas or acts of self-harm or suicide [1–3]. The women’s change into motherhood is a difficult period that involves significant changes in the psychological, social and physiological aspects, and considered increase vulnerability for the development of mental illness [4].

Mental health affects progress towards the achievement of several Sustainable Development Goals (SDGs), such as the promotion of gender equality and empowerment of women, reduction of child mortality and improvement of maternal health. By 2030 one of the goals of SDG to reduce premature mortality by one third from non-communicable diseases through prevention and treatment and promote mental health and wellbeing [5].

About 14% of the global burden of disease has been attributed to neuropsychiatric disorders, mostly due to the chronically disabling nature of depression and other common mental disorders [6]. Eleven percent of the total burden of disease in Ethiopia can be attributed to mental health disorders [7]. More than one in 10 pregnant women and one in 20 postnatal women in Ethiopia suffer from undetected depression [7].

In lifetime, women experienced depression two times more likely than men due to their reproductive nature, caring and rearing of children [8]. Postpartum depression becomes serious public health concern in the developing world and is predicted to become the most common cause of disability by the year 2020 associated with increased mortality through suicide also; it contributes to other associated diseases [9].

It is one of the most common complications of childbearing and is associated with impairments in mother–infant interactions that can lead to severe consequences for the infant such as illness, developmental delay, and poor growth [3, 6, 8]*.* Therefore, this study was aimed to show the prevalence and factors associated with PPD among postpartum mothers live in Ankesha district, Awi Zone, North West Ethiopia.

## Methods

### Study area and period

Community based quantitative cross sectional study was conducted in Ankesha District from February 1 to March 2 / 2018. Ankesha District is one of the Districts in Awi Zone, Amhara regional state of Ethiopia. The District has 31 rural kebeles. The annual report from the Ankesha District office in 2016/17 indicated that the health coverage of the district was 81.4%, institutional delivery 72%, ANC coverage 88%, PNC coverage 58% and immunization coverage 82% [10].

### Source population

All reproductive age group women who gave birth in the last 12 months in Ankesha district.

### Study population

All reproductive age mothers who were living in the selected kebeles and gave birth in the last 12 months.

#### Inclusion criteria

All mothers who gave birth before the interview and residents at least 6 months in the study area.

#### Exclusion criteria

Those mothers who are seriously ill and unable to respond at the time of data collection and mothers who delivered less than 2 weeks before data collection period were excluded from the study.

### Sample size determination

The separate sample size was calculated for each specific objective by using both single and double population proportion formula. The sample size of the first objective was greater than that of the second objective. So the final sample size was come up by adding a non-response rate of 10% to the larger sample size which is 554. Therefore, the calculated sample size for this study was 609. Because of cluster sampling the design effect of 1.5 was added to calculate the sample size for both first and second objectives.

### Data collection method

The questionnaires to assess mother’s socio-demographic characteristics, economic status were adapted from the Ethiopian Demographic and Health Survey (EDHS) 2016. Economic status (wealth index) was computed using principal component analysis (PCA) [11].

Questionnaire to assess depression was assessed by using EPDS. The EPDS is a 10-item self-reporting scale based on a 1 week recall and is specifically designed to screen for PPD. Those women who scored 8 and above were categorized as depressed while women who scored below 8 were considered as none depressed [12]. Social support was measured using the Maternity Social Support Scale (MSSS). The scale contains six items and includes questions on family support, friendship network, and help from a spouse, conflict with a spouse, feeling controlled by a spouse, and feeling unloved by family. Each item measures a five-point Likert scale and a total score of 30 was possible. Social support is classified into three categories; high social support (for scores 24–30), medium social support (18–23) and low social support (below 18). The questionnaire is adapted from previous study on the association between social support and PPD [13]. Substance abuse of both women and husband was assessed by questionnaire adapted from Diagnostic and Statistical Manual of Mental Disorders (DSM-5) criteria used to diagnose substance abuse [14]. A source of information about mental health was also included in the tool.

The data collectors were collected the data through face to face interview.

### Dependent variable

Postpartum depression

### Independent variable

Socio-demographic and economic factors, pregnancy/Obstetric related factors, social support, substance abuse of husband and women, infant sex, mothers’ infant sex preference, previous history of depression, source of information about PPD

### Operational definition

#### Postpartum depressed

Those postpartum mothers who score ≥ 8 cut off point of EPDS. From ten questions each of which has four choices resulting maximum score of 30 and a minimum 0 [15].

#### Normal postpartum (not depressed)

Those mothers who score < 8 cuts off point of EPDS [15]

#### Social support

Social support of the women was measured by MSSS and classified in to three categories;

- ❖ High social support (for scores 24–30)
- ❖ Medium social support (18–23)
- ❖ Low social support (below 18) [13].

### Data quality control

The questionnaire was initially prepared in the English language and then translated into Amharic and local language *“Awigna”* by experts and back-translated to English to check the consistency. The questionnaire was checked for completeness before data entry into software. Proper coding and categorization of data were maintained for the quality of the data to be analyzed. Double data entry was done for its validity and compare to the original data. The pre-test was carried out on 5% of study subjects in one of the kebeles in the District which was not selected as the study kebeles.

### Data processing and analysis

The data was coded, cleaned, edited and entered into Epi data version 3.1 to minimize logical errors, then the data was exported to SPSS window version 24 for analysis. The analysis was done by computing proportions and summary statistics. Then the information was presented by using simple frequencies, tables, pie charts and figures. Bivariate analysis and multivariate analysis was computed to see the association between each independent variable and the outcome variable by using binary logistic regression. The assumptions for binary logistic regression were checked and values below 0.25 in the Bi-variate analysis were considered as candidate variables for multivariate logistic regression [16] to control all possible confounders. The multi co-linearity test was done to see the correlation between independent variables by using the standard error. Hosmer-Lemeshow’s test was found to be insignificant and Omnibus tests were significant which indicates the model was fitted. The direction and strength of statistical association were measured by the odds ratio with 95% CI. The adjusted odds ratio along with 95% CI was estimated to identify factors for PPD by using multivariate analysis in binary logistic regression. In this study *P*-value < 0.05 was considered as statistically significant.

## Result

### Socio-demographic characteristics

In this study, a total of 596 study participants were involved making a response rate of 97.4%. The mean age of the participants was 30.57 (SD ± 6.3) years. More than half 310 of the participants were between the age group of 25–34 years. Half (301) of participants were farmers in their occupation followed by housewives 206 (34.6%). Three forth 441 (74%) of the participant’s husbands were farmers. Two hundred eighteen (36.6%) participant families wealth index were in third quantile (Table 1)*.*

**Table 1:** Socio-demographic/economic characteristics of study participants who delivered in the past 12 months in Ankesha District, North West Ethiopia, 2018 (*n* = 596)

Variables	Frequency	Percentage %
Age		
≤ 24	104	17.4
25–34	310	52
≥ 35	182	30.5
Marital Status		
Married	569	95.5
Others^a^	27	4.5
Occupation of women		
House wife	208	34.9
Merchant	27	4.5
Government employer	38	6.4
Farmer	301	50.5
Daily laborer	22	3.7
Occupation of Husband (569)		
Farmer	426	74.9
Merchant	51	9.1
Student	33	5.8
Government employer	15	2.6
Daily laborer	32	5.6
Other^b^	12	2.1
Wealth Index		
Third Quantile	218	36.6
Second Quantile	187	31.4
First Quantile	191	32.0

Others^a^ = divorced, widowed, unmarried

Other^b^ = priests, jobless

A larger proportion of participants 573 (96.1%) claimed they were Ortodox Christianity religion followers, whereas protestant religion consittuents 16 (3%) and other like traditional 7 (1.2%). Of the participants about two-third, 404 (67.8%) were no formal education followed by primary education 154 (25.8%) and secondary and above 38 (6.4%). From husbands of participants, more than three forth 444 (78%) have no formal education followed by primary, secondary and above with magnitude of 95 (16.7%) and 30 (5.3%) respectively.

### Obstetrics characteristics

Two third (389) of participants were multiparous and 505 (84%) have two or more alive children. From the participants, 487 (81.7%) have no history of abortion. More than three fourth 475 of study participants replied that their current pregancny was wanted, and 446 (74.8%) participants attended ANC follow up at least once and 173 (38.8%) had four ANC follow up. Among 446 ANC attendee participants, 397 (89%) of them had not counseled about PPD. Thirty nine percent (234) of study participants was attended pregnant mothers monthly meeting during pregnancy, from those who attended the meeting only 15(6.4%) was discussed about PPD.

Nearly two-thirds of 373 (62.6%) were delivered by SVD. From participants, one forth (149) was given birth at home. Mothers who deliver at institution 258 (57.7%) was stayed less or equal to 1 day at institution. More than half 321 of the participant’s current infants were females. For 334 (56%) women the desired sex was male and 319 (53.5%) of the mother’s sex preference was not meet. Thirty-one percent (185) of infants were ill and from those, more than two-third 131 (70.8%) were treated by inpatient or outpatient. Of the participants, 111 (18.6%) was the previous history of neonatal loss. One hundred eleven (18.6%) participants were PNC follow up from those more than three forth 94 (85%) were not counseled about PPD. Postpartum home care was given by family for most 411 (69%) of participants followed by HEWs 105 (17.6%) and neighbors 80 (13.4%) (Table 2).

**Table 2:** Obstetric characteristics of study participants who delivered in the past 12 months in Ankesha District, North West Ethiopia, 2018 (*n* = 596)

Variables	Classification	Post partum Depression		Frequency (%)	Chi square
		Depressed (%)	Not Depressed (%)		
Parity	Primiparus	32 (22.7)	43 (9.5)	75 (12.6)	
	Multiparus	79 (56)	310 (68.1)	389 (65.3)	0.00
	Grand Multiparus	30 (21.3)	102 (22.4)	132 (22.1)	
No of alive child	≤1	37 (26.2)	54 (11.9)	91 (15.3)	0.00
	≥2	104 (73.8)	401 (88.1)	505 (84.7)	
Counseled About PPD (*n* = 446)	Yes	16 (14.4)	33 (9.9)	49 (11.0%)	
	No	95 (85.6)	302 (90.1)	397 (89%)	0.2
Mode of Delivery	SVD	92 (65.2)	281 (61.8)	373 (62.6)	
	Instrumental	34 (24.1)	127 (27.9)	161 (27)	0.67
	Operation	15 (10.7)	47 (10.3)	62 (10.4)	
Assisting delivery	health worker	106 (75.2)	352 (77.4)	458 (76.9)	
	Traditional	15 (10.6)	37 (8.1)	52 (8.7)	0.68
	Family	20 (14.2)	66 (14.5)	86 (14.4)	
Length of stay in days at institution (447)	1	52 (55.9)	206 (58.2)	258 (57.7)	
	≥2	41 (44.1)	148 (41.8)	189 (42.3)	0.72
Delivery complication	Yes	58 (41.4)	108 (23.6)	166 (27.9)	0.00
	No	83 (58.6)	347 (76.4)	430 (72.1)	
Desired sex of you	Male	76 (53.9)	258 (56.7)	334 (56)	0.56
	Female	65 (46.1)	197 (43.3)	262 (44)	
Sex preferance of mother	Non preference	93 (66)	226 (49.7)	319 (53.5)	0.001
	Preferance	48 (44)	229 (50.3)	277 (46.5)	
Index infant illness	Yes	70 (49.6)	115 (25.3)	185 (31.04)	0.000
	No	71 (50.4)	340 (74.7)	411 (68.9)	

### Previous history of depression

Almost all 581 (97.5%) had no history of mental illness. From the participants, more than three forth 549 (91%) had no previous history of depression. Five hundred seven (85%) of study participants had no family history of mental illness.

### Substance abuse and social support

From the total study participants, almost all 578 (97%) were not substance abused. The husbands of participants 497 (87.4%) were not substance abused*.*

**Fig. 1:**
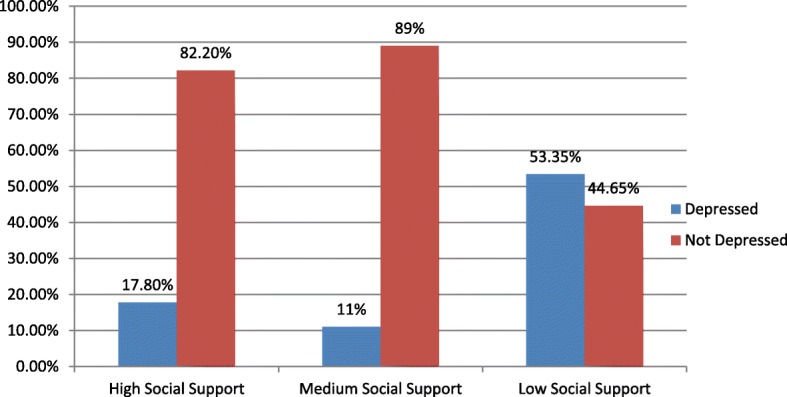
Social support of study participants who delivered in the past 12 months in Ankesha District, North West Ethiopia, 2018 (*n* = 596)

More than half 316 (53%) of participants social support was medium while 74 (12.4%) was high (Fig. 1).

### Prevalence of postpartum depression

In this study the proportion of women who had PPD was 141 (23.7%) with 95% CI: 20.3–27.2. Mean score of 6.69, (Std. Deviation ±4.33), Minimum cumulative score 0 (1), Maximum cumulative score 22 (1). Most of respondents 141 (23.7%) score 5 followed by 99 (16.6%) score 6.

### Predictors of postpartum depression

Variables that fulfill the criteria in Bivariate analysis were marital status, parity, number of alive babies, unwanted pregnancy, delivery complication, mothers’ preference for the infant’s gender, illness of the infant, previous neonatal loss, previous history of depression, husbands substance abuse and social support. These variables were enterd in to a multivariate logistic regression model from that, marital status, unwanted pregnancy, mothers’s preference for the infant’s gender, infant illness and social support were statistically associated with PPD. Divorced/widowed/single participants were 3.45 times more likely to develop PPD than married (AOR = 3.45 95%CI: 1.35–8.82). Mothers whose preganacies were unwanted (AOR = 1.95 95%CI: 1.14–3.33) and those who got infant of unpreferred sex (AOR = 1.79 95%CI: 1.13–2.86) were found to be significant factors for postpartum depression.

Respondents whose baby was ill before data collection were two times more likely depressed than those who were not ill (AOR = 2.08 95% CI: 1.30–3.34). Mothers who had a previous history of depression were 3.7 times more likely depressed than their counterparts (COR = 3.73 95%CI: 2.086–6.67). Those participants with low social support were 3.16 times more likely depressed than those who had high social support (AOR = 3.16 95%CI: 1.55–6.43) (Table 3).

**Table 3:** Predictors of postpartum depression among mothers who delivered in the past 12 months in Ankesha District, North West Ethiopia, 2018 (*n* = 596)

Variables	Post partum depression		(95% CI)	
	Depressed %	Not depressed %	Crude OR	Adjusted OR
Marital status				
Married	127 (90.1)	442 (97.1)	1	1
Others	14 (9.9)	13 (2.9)	3.74 (1.71–8.17)	3.45 (1.35–8.82)^*^
Parity				
Primiparus	32 (22.7)	43 (9.5)	2.53 (1.37–4.66)	2.68 (0.62–11.56)
Multiparus	79 (56)	310 (68.1)	0.86 (0.53–1.39)	1. 04 (0.59–1.80)
Grand multiparus	30 (21.3)	102 (22.4)	1	1
No of alive baby				
≤ 1	37 (26.2)	54 (11.9)	2.64 (1.65–4.23)	0.8 (0.22–2.90)
≥ 2	104 (73.8)	401 (88.1)	1	1
Wanted pregnancy				
Yes	98 (69.5)	377 (82.9)	1	1
No	43 (30.5)	78 (17.1)	2.12 (1.37–3.27)	1.95 (1.14–3.33)^**^
Delivery complication				
Yes	58 (41.4)	108 (23.6)	2.25 (1.53–3.42)	1.24 (0.74–2.08)
No	83 (58.6)	347 (76.4)	1	1
Sex preference of mother				
Non preferred	93 (66)	226 (49.7)	1.96 (1.32–2.91)	1.79 (1.13–2.86)***
Preferred	48 (44)	229 (50.3)	1	1
Last infant illness				
Yes	70 (49.6)	115 (25.3)	2.91 (1.97–4.31)	2.08 (1.30–3.34)****
No	71 (50.4)	340 (74.7)	1	1
Neonatal loss				
Yes	41 (29.1)	70 (15.4)	2.25 (1.44–3.51)	1.50 (0.85–2.66)
No	100 (70.9)	385 (84.6)	1	1
Previous history of depression				
Yes	26 (18.4)	26 (5.7)	3.73 (2.08–6.67)	1.96 (0.97–3.96)
No	115 (81.6)	429 (94.3)	1	1
Husband substance abuse				
None abused	108 (76.6)	389 (85.5)	1	1
Abused	33 (23.4)	66 (14.5)	1.80 (1.12–2.87)	1.09 (0.61–1.96)
Social support				
High	14 (9.9)	60 (13.2)	1	1
Medium	35 (24.8)	281 (61.7)	0.53 (0.27–1.05)	0.73 (0.34–1.55)
Low	92 (65.3)	114 (25.1)	3.45 (1.81–6.58)	3.16 (1.55–6.43)*****

*Significant with *P* = 0.01, **Significant with *P* = 0.014, ***Significant with *P* = 0.014, ****Significant with *P* = 0.002 and *****Significant with *P* = 0.001

## Discussion

In this study, the prevalence of PPD was 23.7% (95% CI: 20.3–27.2). Factors like marital status, prim parity, unwanted pregnancy, delivery complication, number of live babies, unpreferred infant sex by the mother, infant illness, previous infant loss, previous history of depression, substance abuse of husband and social support were associated with PPD in Bivariate analysis. Marital status, unwanted pregnancy, unmet sex preference of the mother, infant illness and social support were independently associated with PPD.

In this study, nearly one-forth of the study participants suffered from postpartum depression. This finding was in line with the studies conducted in Pakistan (23%), Bangladesh (22%), North Gonder (24.1%) and Bahir Dar (21.5%) [17–19]. The result was slightly higher than studies conducted in Argentina (18.6%), Kenya (20%), Egypt (7.1%), Malawi (11%), Butajira (12.7%) and Eastern tigray (19%) [9, 16, 20–23]. But it was lower than studies conducted in China (27.37%), Basra (31.5%), India (48.5%) and Zimbabwe (33%) [24–26]. The possible reason for the variation of the result may be the difference in assessment tool and cut off point values used to classify mothers as depressed and not depressed. Population differences also may contribute to the variation because some studies were done in urban settings. Besides, it could also be due to the methodological difference, some of the studies used institution based study design with low sample size.

(Divorced/widowed/ unmarried) women were 3.45 times more likely to develop PPD than married. Possibly, those women are prone to social, economic, and psychological challenges, which in turn may aggravate the condition of depression. It may also be the fact that the issue of adverse life events of losing someone they like most, then both economical and social loss follows. This is inconsistent with studies conducted in Kenya and three zones of the Amhara region, Ethiopia [19, 27].

Mothers who had unwanted pregnancy were two times more likely to be depressed than women whose pregnancy was wanted. This is in line with studies conducted in Mexico, India, India Bangalore, Egypt [21, 26, 28, 29]. The reason may be due to pregnancy in itself is a major experience in women’s life, So it demands physiological, psychological, social adjustments and financial preparation. The social and economic burden resulting from unplanned pregnancies for which adequate preparation was not made might result in psychological distress. Also in our setting unwanted pregnancy is mostly associated with economical status and this can be lead to worrying for parents’ and the coming babies’ basic needs and better quality life.

The women whose infant sex not preferred by the mother were 1.75 times more likely to develop depression than those infant sex preferred. This finding is in line with studies conducted in Basra, Mexico, Kenya [25, 28, 30]. The reason may be due to the preferred sex of the mother is mostly preferred sex of the family as a whole, So if this is not meet there may be social isolation lead to stress and depression.

Respondents whose baby was ill before data collection were two times more likely depressed than those who were not ill. This finding is in line with studies done in India [26]. This might be since negative life events are most influential on an individual’s mental status. It also might be because they frightened about the lose of their infant. Also, economically payment for the baby’s treatment and if there is admission there is overcrowding of health institutions that may make the mother anxious and depressed.

Those participants with low social support were 3.3 times more likely depressed than those who had high social support. This finding agreed with many studies conducted in different areas like India, Arab, Sudan, two studies in Ethiopia [16, 18, 31–33]. The reason may be due to that not having social support makes them vulnerable to stress, loneliness and hopelessness. Also, those women who received a partner’s support during their postpartum period will empower them to deal with their home responsibility. In addition, the fact that social support plays a buffering role from stressful life events by providing resources, support, and strength during postpartum period.

As this study indicates socio-demographic factors like age, educational status, occupation and economic status of the mother were not significantly associated with PPD. This is contradicted with studies done in Basra, Iran, India [29, 31, 34]. The reason may be due to demographical and socio-cultural differences. Obstetric factors like complications during delivery, mode of delivery by cesarean section were not significantly associated in this study. This is incongruent with the study done in Argentina and Egypt [20, 21]. This may be because there are some improvements in the health care systems on maternal health. Substance abuse of both women and husband were not significantly associated with PPD in this study. The finding differs from studies done in Canada, Mexico, Northern India, Bale zones, Oromia region of Ethiopia [12, 20, 28, 31, 35]. The reason may be due to culture protects the participants and their husbands from substance abuse.

Due to social isolation and stigma towards mental illness respondents might not respond correctly. The study might be subjected to recall bias because the mothers failed to remember previous conditions. Because of the cross-sectional study design, the study might not show cause and effect relationships.

## Conclusion

One in five women in the study area suffers from postpartum depression. This sparkes light to health professionals to pay attention to the prevention and treatment of postpartum depression. Marital status, unwanted pregnancy, unpreferred infant sex by the mother, infant illness and poor social support were independently associated with postpartum depression.

## Supplementary information

**Additional file 1.** Questionnaire.

## Data Availability

The datasets used and/or analyzed during the current study available from the corresponding author on reasonable request.

## Abbreviations

CI: Confidence Interval
CSA: Central Statistical Agency
EDHS: Ethiopian Demographic Health Survey
EPDS: Eden Burg Post Partum Depression Scale
HU: Haramaya University
IEOS: Integrated Emergency Obstetric and Surgery
IHRERC: Institutional Health Research Ethics Review Committee
LMIC: Lower and Middle Income Countries
MSSS: Maternity Social Support Scale
PI: Principal Investigator
PPD: Post Partum Depression
SDG: Sustainable Development Goal
SPSS: Statistical Package for Social Science
WHO: World Health Organization
