# Supplementary material for: Postpartum depression and associated factors among mothers who gave birth in the last twelve months in Ankesha district, Awi zone, North West Ethiopia
Source: BMC Pregnancy Childbirth. 2019 Nov 21;19:435. doi: 10.1186/s12884-019-2594-y (PMC6873414; doi:10.1186/s12884-019-2594-y)
Supplement: Supplementary file 1 — Additional file 1. Questionnaire. [file 12884_2019_2594_MOESM1_ESM.docx]

**Questionnaire**

As indicated at the methods part of data collection methods the questionnaire was developed from different study literature and the English version questionnaire is below.

**This is the questionnaire to assess the Prevalence of PPD and associated factors among Mothers who gave birth in the last twelve months, Ankesha district, Awi zone, Ethiopia, 2018**

01. Keble: ____________________

02. House code__________________

03. Code of the Women__________________

03. Name of Data Collector: ____________ Signature__________ Date: _______

04. Name of Supervisor: ___________ ______ Signature_______ Date: _____**__**

| \|  \| \| --- \|  \|  \| \| \| \| \| \| \| \| \| \| --- \| --- \| --- \| --- \| --- \| --- \| --- \| --- \| --- \| \| **S NO** \| \| **Question** \| \| **Response** \| \| \| **Skip** \| \| \| **PART I: SOCIO-DEMOGRAPHIC CHARACTERISTICS** \| \| \| \| \| \| \| \| \| \| 101 \| Age of the mother \| \| /_______/ in complete year \| \| \| \|  \| \| \| 102 \| Marital status \| \| 1. Married  2. Single  3. Divorced  4. Widowed \| \| \| \|  \| \| \| 103 \| Religion \| \| 1. Orthodox  2. Protestant  3. Catholic  4. Muslim  5. Traditional  6. If other specify................ \| \| \| \|  \| \| \| 104 \| Educational status of the mother \| \| 1. Cannot able to read and write  2. Can read and write  3. Grade 1-8  4. Grade 9-12  5. College and above \| \| \| \|  \| \| \| 105 \| Occupation of the mother \| \| 1. Housewife  2. Merchant  3. Government employer  4. Farmer  5. Daily laborer  6. Student  7. Other, Specify_______ \| \| \| \|  \| \| \| 106 \| Educational status of husband \| \| 1. Cannot able to read and write  2. Can read and write  3. Grade 1-8  4. Grade 9-12  5. College and above \| \| \| \|  \| \| \| 107 \| Husband’s occupational status \| \| 1. Farmer  2. Merchant  3. Student  4. Civil servant  5. Day laborer  6. Others specify \| \| \| \|  \| \| \| **PART II: SOCIO-ECONOMIC STATUS/ WEALTH INDEX QUESTIONS** \| \| \| \| \| \| \| \| \| \| 201 \| Does any member of this household has owned any agricultural land? \| \| 1. Yes  2. No \| \| \| \| If “No” skip to Q202 \| \| \| 202 \| How much (local units) of agricultural land do members of this household own? \| \| _____________( in Hectare) \| \| \| \|  \| \| \| 203 \| Is the land cultivated? \| \| 1. Yes  2. No \| \| \| \| If “No” skip to Q204 \| \| \| 204 \| How much land is cultivated (local unit)? \| \| _____(in Hectare) \| \| \| \|  \| \| \| 205 \| Does your family have any stored grains/cereals in the house? \| \| 1. Yes  2. No \| \| \| \| If “No” skip to Q206 \| \| \| 206 \| How many “kunital”? \| \| _______(in number) \| \| \| \|  \| \| \| 207 \| Does this household own any livestock, herds, other farm animals, or poultry? \| \| 1. Yes  2. No \| \| \| \| If “No” skip to Q208 \| \| \| 208 \| How many of the following animals, does this household own? \| \| \| Animals \| No \| \| --- \| --- \| \| Milk cows, oxen or bulls \|  \| \| Horses, donkey or mules \|  \| \| Got \|  \| \| Sheep \|  \| \| Chicken \|  \| \| Beehives \|  \| \| \| \| \|  \| \| \| 209 \| Which of the following does your household have? *(Record observation)*  (*Multiple responses is possible)* \| \| 1. Electricity  2. Watch  3. Radio  4. Television  5. Mobile Telephone  6. Non-Mobile Telephone  7. Chair  8. Table  9. Bed  10. Electric Mitad  11. Other (specify)_______ \| \| \| \|  \| \| \| 210 \| What is the main current source of drinking water for members of your household? \| \| 1. Piped water  2. Public Tap/Stand Pipe  3. Borehole  4. Protected well  5. Unprotected well  6. Protected Spring  7. Unprotected Spring  8. River/Ponds/Stream/Dam  9. Other (specify) ______ \| \| \| \|  \| \| \| 211 \| Do you do anything to the drinking water to make it safer to drink? \| \| 1. Yes  2. No \| \| \| \|  \| \| \| 212 \| What is the main source of water used by your household for other purposes such as cooking and handwashing? \| \| 1. Piped water  2. Public Tap/Stand Pipe  3. Borehole  4. Protected well  5. Unprotected well  6. Protected Spring  7. Unprotected Spring  8. River/Ponds/Stream/Dam  9. Other (specify)_______ \| \| \| \|  \| \| \| 213 \| What kind of toilet facility do members of your household usually use?  *(Record Observation)* \| \| 1. Flush to a piped sewer system  2. Flush to a septic tank  3. Pit latrine with slab  4. Pit latrine without a slab  5. Ventilated improved pit latrine  6. No facility/bush/field  7. Other (specify)_____ \| \| \| \|  \| \| \| 214 \| What are the main materials of the floor of the house?  *(Record observation)* \| \| 1. Earth/Sand  2. Wood planks  3. Palm/Bamboo  4. Ceramic Tiles  5. Cement  6. Other (specify)______ \| \| \| \|  \| \| \| 215 \| What are the main materials of the roof of the house?  *(Record observation)* \| \| 1. Thatch/straw  2. Leaf/Earth/ Mud/Cow dung  3. Wood planks, cardboard  4. Finished roof (iron, tin, finished wood, cement, ceramic)  5. Other(specify)___________ \| \| \| \|  \| \| \| 216 \| What is the main material of the exterior walls of the house?  *(Record observation)* \| \| 1. Simple wall with mud or local materials  2. Bamboo or stone with mud, plywood, cardboard  3. Finished walls; cement, brick, stone with cement, wood planks  4. No outside walls  5. Others (specify)______ \| \| \| \|  \| \| \| 217 \| How many members are live in your household? \| \| ___________(in number) \| \| \| \|  \| \| \| 218 \| How many rooms are there for your house? \| \| ___________(in number) \| \| \| \|  \| \| \| 219 \| Do you have a separate room which is used as a kitchen? *(Record observation)* \| \| 1. Yes  2. No \| \| \| \|  \| \| \| 220 \| What type of fuel do you mainly use for cooking? \| \| 1. Electricity  2. Wood  3. Kerosene  4. Animal dung  5. Charcoal  6. Shrubs/Grass  7. Crops  8. Other (specify)_____ \| \| \| \|  \| \| \| 221 \| Which means of transport (vehicles) does any member of your household have? \| \| 1. Bicycle  2. Motor cycle  3. Animal-drawn cart  4. Car/truck  5. None \| \| \| \|  \| \| \| 222 \| Does any member of this household have a bank or micro-finance savings account? \| \| 1. Yes  2. No \| \| \| \|  \| \| \| 223 \| How much of money? \| \| ___________(Ethiopian Birr \| \| \| \| \| \| \| **PART III: OBSTETRIC RELATED QUESTIONS** \| \| \| \| \| \| \| \| \| \| 301 \| How many times do you gate pregnant? \| \| ------------------ \| \| \| \|  \| \| \| 302 \| How many times did you give birth? \| \| ------------------ \| \| \| \|  \| \| \| 303 \| Of these pregnancies how many terminated before 7 months (28 weeks)? \| \|  \| \| \| \|  \| \| \| 304 \| Is the pregnancy wanted and planned? \| \| 1. Yes  2. No \| \| \| \|  \| \| \| 305 \| Do you have ANC visit in this delivery during pregnancy? \| \| 1. Yes  2. No \| \| \| \| If “No” Skip to Q406 \| \| \| 306 \| How many times did you receive ANC service from health care providers?) \| \| ___________(in number \| \| \| \|  \| \| \| 307 \| Do you have any medical disorder during pregnancy \| \| 1. Yes  2. No \| \| \| \|  \| \| \| 308 \| If Yes, Have you admitted to a health institution? \| \| 1. Yes  2. No \| \| \| \|  \| \| \| 309 \| Have you advised about PPD during ANC visits? \| \| 1. Yes  2. No \| \| \| \|  \| \| \| 310 \| Have you attended a monthly pregnant mother's group meeting? \| \| 1. Yes  2. No \| \| \| \|  \| \| \| 311 \| Have you discussed PPD during the monthly pregnant mother's meeting? \| \| 1. Yes  2. No \| \| \| \| If “No” Skip to  Q305 \| \| \| 312 \| Where you gave birth? \| \| 1. Health Center  2. Hospital  3. Health Post  4. Home \| \| \| \|  \| \| \| 313 \| Who attended/ assisted the delivery? \| \| 1. Health care provider  2. Family  3. Neighbor  4. Relatives(mother in law)  5. Traditional Birth Attendant (TBA)  6. Health Extension Worker (HEW)  7. Other, Specify________________ \| \| \| \|  \| \| \| 314 \| In which mode of delivery you gave birth? \| \| 1. Spontaneous vaginal delivery  2. Instrumental assisted delivery  3. Cesarean section \| \| \| \| For institutional delivery only \| \| \| 315 \| Had you faced any type of complication during the delivery? \| \| 1. Yes  2. No \| \| \| \|  \| \| \| 316 \| How long you stayed at health institution \| \| _____________ \| \| \| \|  \| \| \| 317 \| Are you on any medication since you delivered? \| \| 1. Yes specify ______________  2. No \| \| \| \|  \| \| \| 318 \| What is the sex of the baby \| \| 1. Male  2. Female \| \| \| \|  \| \| \| 319 \| What was the desired sex of you \| \| 1. Male  2. Female \| \| \| \|  \| \| \| 320 \| Does your child become ill? \| \| 1. Yes  2. No \| \| \| \|  \| \| \| 321 \| If yes, How the illness treated? \| \| 1. None  2. Outpatient  3. Admitted \| \| \| \|  \| \| \| 322 \| Do you have a neonatal loss in the previous delivery? \| \| 1. Yes  2. No \| \| \| \|  \| \| \| 323 \| Who gave care to you after delivery? \| \| 1. Health Extension Worker (HEW)  2. Family/ mother in law  3. Neighbor  4. My mom  5. Other, Specify_______________ \| \| \| \|  \| \| \| 324 \| Do you have an immediate PNC visit? \| \| 1. Yes  2. No \| \| \| \| If No skip 325 \| \| \| 325 \| Do you advise about PPD? \| \| 1. Yes  2. No \| \| \| \|  \| \| \| **PART FOUR:-- EDINBURGH POSTNATAL DEPRESSION SCALE (EPDS) IN THE PAST 7 DAYS**  As you have recently had a baby, we would like to know how you are feeling now and **the past seven days not just how you feel today**.  For example, I have felt happy:  Yes, all the time  Yes, most of the time  No, not very much  No, not at all  This would mean: ‘I have felt happy most of the time during the past week.’ **In the past seven days.** \| \| \| \| \| \| \| \| \| \| 401 \| In the past seven days have u ever experienced laugh and see the funny side of things? \| \| 1. As much as I always could  2. Not quite so much now  3. Not so much now  4. Not at all \| \| \| \|  \| \| \| 402 \| In the past seven days have u ever looked forward with enjoyment to things? \| \| 1. As much as I ever did  2. Rather less than I used to  3. Less than I used to  4. Hardly not at all \| \| \| \|  \| \| \| 403 \| In the past seven days have you blamed yourself unnecessarily when things went wrong? \| \| 1. No, never  2. Not very often  3. Yes, some of the time  4. Yes, most of the time \| \| \| \|  \| \| \| 404 \| In the past seven days have you ever been anxious or worried for no good reason? \| \| 1. No, not at all  2. Hardly ever  3. Yes, sometimes  4. Yes, very often \| \| \| \|  \| \| \| 405 \| In the past seven days have you felt scared or panicky for no very good reason? \| \| 1. No, not at all  2. No, not much-  3. Yes, sometimes  4. Yes, quite a lot \| \| \| \|  \| \| \| 406 \| In the past seven days, things have been getting on top of you? \| \| 1. No, I have been coping as well as ever  2. No, most of the time I have coped quite well  3. Yes, sometimes I haven’t been coping as well as usual  4. Yes, most of the time I haven’t been able to cope at all \| \| \| \|  \| \| \| 407 \| In the past seven days have you been so unhappy that you have had difficulty sleeping? \| \| 1. No, not at all  2. Not very often  3. Yes, sometimes  4. Yes, most of the time \| \| \| \|  \| \| \| 408 \| In the past seven days have you felt sad or miserable? \| \| 1. No, not at all  2. Not very often  3. Yes, quite often  4. Yes, most of the time \| \| \| \|  \| \| \| 409 \| In the past seven days have you been so unhappy that you have been crying? \| \| 1. No, never -  2. Only occasionally  3. Yes, quite often  4. Yes, most of the time \| \| \| \|  \| \| \| 410 \| In the past seven days did you have the thought of harming yourself? \| \| 1. Never  2. Hardly ever  3. Sometimes  4. Yes, quite often \| \| \| \|  \| \| \| **PART V SOURCE OF INFORMATION ABOUT PPD** \| \| \| \| \| \| \| \| \| \| 501 \| Have you ever heard about PPD \| \| 1. Yes  2. No \| \| \| \| If No skip 302 \| \| \| 502 \| About What?  *(Multiple responses is possible)* \| \| 1. About the cause of PPD  2. The occurrence of PPD  3. The definition of PPD \| \| \| \|  \| \| \| 503 \| From which source you heard the information?  *(Multiple responses is possible)* \| \| 1. Health professionals  2. Health Extension Workers (HEWs)  3. Health Development Army (HDA)  4. Neighbors  5. Media  6.Other, Specify________________ \| \| \| \|  \| \| \| **PART VI PREVIOUS HISTORY OF DEPRESSION** \| \| \| \| \| \| \| \| \| \| 601 \| Have you ever have a history of mental illness \| \| 1. Yes  2. No \| \| \| \|  \| \| \| 602 \| If yes when \| \| 1. Before pregnancy  2. During pregnancy  3. After delivery \| \| \| \|  \| \| \| 603 \| Do you have a history of depression in the previous deliveries \| \| 1. Yes  2. No \| \| \| \|  \| \| \| 604 \| Do you have a family history of mental illness \| \| 1. Yes  2. No \| \| \| \|  \| \| \| **PART VII SUBSTANCE USE** \| \| \| \| \| \| \| \| \| \| 701 \| Have you ever used Alcohol (beer) before pregnancy? \| \| 1. Yes  2. No \| \| \| \| If No skip to 702 \| \| \| 702 \| How many times \| \| 1. Tried once  2. Used 1 times /month  3. Used 1 times /week  4. >1 times /week  5. Used daily \| \| \| \|  \| \| \| 703 \| Have you used Alcohol in your last delivery? \| \| 1. Yes  2. No \| \| \| \| If No skip to 704 \| \| \| 704 \| How many times \| \| 1. Tried once  2. Used 1 times /month  3. Used 1 times /week  4. >1 times /week  5. Used daily \| \| \| \|  \| \| \| 705 \| Have you ever used cigarettes before pregnancy? \| \| 1. Yes  2. No \| \| \| \| If No skip to 706 \| \| \| 706 \| How many times \| \| 1. Tried once  2. Used 1 times /month  3. Used 1 times /week  4. >1 times /week  5. Used daily  6. More than once daily \| \| \| \|  \| \| \| 707 \| Have you used a Cigarette in your last delivery? \| \| 1. Yes  2. No \| \| \| \| If No skip to 708 \| \| \| 708 \| How many times \| \| 1. Tried once  2. Used 1 times /month  3. Used 1 times /week  4. >1 times /week  5. Used daily  6. More than once daily \| \| \| \|  \| \| \| 709 \| Did your husband use Alcohol (beer)? \| \| 1. Yes  2. No \| \| \| \| If No skip to 710 \| \| \| 710 \| How many times \| \| 1. Tried once  2. Used 1 times /month  3. Used 1 times /week  4. >1 times /week  5. Used daily \| \| \| \|  \| \| \| 711 \| Did your husband used Cigarette \| \| 1. Yes  2. No \| \| \| \| If No skip to 710 \| \| \| 712 \| How many times \| \| 1. Tried once  2. Used 1 times /month  3. Used 1 times /week  4. >1 times /week  5. Used daily  6. More than once daily \| \| \| \|  \| \| \| **PART VIII SOCIAL SUPPORT** \| \| \| \| \| \| \| \| \| \|  \|  \| \| 1. Strongly agree \| \| 2. Agree \| 3.Undecided \| 4. Disagree \| 5. Strongly disagree \| \| 801 \| Whenever you need help, you ask your family for support? \| \|  \| \|  \|  \|  \|  \| \| 802 \| Do you agree your family friendship network is good? \| \|  \| \|  \|  \|  \|  \| \| 803 \| Do you agree your spouse assured you that you can rely completely on him? \| \|  \| \|  \|  \|  \|  \| \| 804 \| Do you have a conflict with a spouse in the past days? \| \|  \| \|  \|  \|  \|  \| \| 805 \| Do you agree you are feeling controlled by family? \| \|  \| \|  \|  \|  \|  \| \| 806 \| Do you agree your family showed you that they love and accept you? \| \|  \| \|  \|  \|  \|  \| |
| --- | --- | --- | --- | --- | --- | --- | --- | --- | --- | --- | --- | --- | --- | --- | --- | --- | --- | --- | --- | --- | --- | --- | --- | --- | --- | --- | --- | --- | --- | --- | --- | --- | --- | --- | --- | --- | --- | --- | --- | --- | --- | --- | --- | --- | --- | --- | --- | --- | --- | --- | --- | --- | --- | --- | --- | --- | --- | --- | --- | --- | --- | --- | --- | --- | --- | --- | --- | --- | --- | --- | --- | --- | --- | --- | --- | --- | --- | --- | --- | --- | --- | --- | --- | --- | --- | --- | --- | --- | --- | --- | --- | --- | --- | --- | --- | --- | --- | --- | --- | --- | --- | --- | --- | --- | --- | --- | --- | --- | --- | --- | --- | --- | --- | --- | --- | --- | --- | --- | --- | --- | --- | --- | --- | --- | --- | --- | --- | --- | --- | --- | --- | --- | --- | --- | --- | --- | --- | --- | --- | --- | --- | --- | --- | --- | --- | --- | --- | --- | --- | --- | --- | --- | --- | --- | --- | --- | --- | --- | --- | --- | --- | --- | --- | --- | --- | --- | --- | --- | --- | --- | --- | --- | --- | --- | --- | --- | --- | --- | --- | --- | --- | --- | --- | --- | --- | --- | --- | --- | --- | --- | --- | --- | --- | --- | --- | --- | --- | --- | --- | --- | --- | --- | --- | --- | --- | --- | --- | --- | --- | --- | --- | --- | --- | --- | --- | --- | --- | --- | --- | --- | --- | --- | --- | --- | --- | --- | --- | --- | --- | --- | --- | --- | --- | --- | --- | --- | --- | --- | --- | --- | --- | --- | --- | --- | --- | --- | --- | --- | --- | --- | --- | --- | --- | --- | --- | --- | --- | --- | --- | --- | --- | --- | --- | --- | --- | --- | --- | --- | --- | --- | --- | --- | --- | --- | --- | --- | --- | --- | --- | --- | --- | --- | --- | --- | --- | --- | --- | --- | --- | --- | --- | --- | --- | --- | --- | --- | --- | --- | --- | --- | --- | --- | --- | --- | --- | --- | --- | --- | --- | --- | --- | --- | --- | --- | --- | --- | --- | --- | --- | --- | --- | --- | --- | --- | --- | --- | --- | --- | --- | --- | --- | --- | --- | --- | --- | --- | --- | --- | --- | --- | --- | --- | --- | --- | --- | --- | --- | --- | --- | --- | --- | --- | --- | --- | --- | --- | --- | --- | --- | --- | --- | --- | --- | --- | --- | --- | --- | --- | --- | --- | --- | --- | --- | --- | --- | --- | --- | --- | --- | --- | --- | --- | --- | --- | --- | --- | --- | --- | --- | --- | --- | --- | --- | --- | --- | --- | --- | --- | --- | --- | --- | --- | --- | --- | --- | --- | --- | --- | --- | --- | --- | --- | --- | --- | --- | --- | --- | --- | --- | --- | --- | --- | --- | --- | --- | --- | --- | --- | --- | --- | --- | --- | --- | --- | --- | --- | --- | --- | --- | --- | --- | --- | --- | --- | --- | --- | --- | --- | --- | --- | --- | --- | --- | --- | --- | --- | --- | --- | --- | --- | --- | --- | --- | --- | --- | --- | --- | --- | --- | --- | --- | --- | --- | --- | --- | --- | --- | --- | --- | --- | --- | --- | --- | --- | --- | --- | --- | --- | --- | --- | --- | --- | --- | --- | --- | --- | --- | --- | --- | --- | --- | --- | --- | --- | --- | --- | --- | --- | --- | --- | --- | --- | --- | --- | --- | --- | --- | --- | --- | --- | --- | --- | --- | --- | --- | --- | --- | --- | --- | --- | --- | --- | --- | --- | --- | --- | --- | --- | --- | --- | --- | --- | --- | --- | --- | --- | --- | --- | --- | --- | --- | --- | --- | --- | --- | --- | --- | --- | --- | --- | --- | --- | --- | --- | --- | --- | --- | --- | --- | --- | --- | --- | --- | --- | --- | --- | --- | --- | --- | --- | --- | --- | --- | --- | --- | --- | --- | --- | --- | --- | --- | --- | --- | --- | --- | --- | --- | --- | --- | --- | --- | --- | --- | --- | --- | --- | --- | --- | --- | --- | --- | --- | --- | --- | --- | --- | --- | --- | --- | --- | --- | --- | --- | --- | --- | --- | --- | --- | --- | --- | --- | --- | --- | --- | --- | --- | --- | --- | --- | --- | --- | --- | --- | --- | --- | --- | --- | --- | --- | --- | --- | --- | --- | --- | --- | --- | --- | --- | --- | --- | --- | --- | --- | --- | --- | --- | --- | --- | --- | --- | --- | --- | --- | --- | --- | --- | --- | --- | --- | --- | --- | --- | --- | --- | --- | --- | --- | --- | --- | --- | --- | --- | --- | --- | --- | --- | --- | --- | --- | --- | --- | --- | --- | --- | --- | --- | --- | --- | --- | --- | --- | --- | --- | --- | --- | --- | --- | --- | --- | --- | --- | --- | --- | --- | --- | --- | --- | --- | --- | --- | --- | --- | --- | --- | --- | --- | --- | --- | --- | --- | --- | --- | --- | --- | --- | --- | --- | --- | --- | --- | --- | --- | --- | --- | --- | --- | --- | --- | --- | --- | --- | --- | --- | --- | --- | --- | --- | --- | --- | --- | --- | --- | --- | --- | --- | --- | --- | --- | --- | --- | --- | --- | --- | --- | --- | --- | --- | --- | --- | --- | --- | --- | --- | --- | --- | --- | --- | --- | --- | --- | --- | --- | --- | --- | --- | --- | --- | --- | --- | --- | --- | --- | --- | --- | --- | --- | --- | --- | --- | --- | --- | --- | --- | --- | --- | --- | --- | --- | --- | --- | --- | --- | --- | --- | --- | --- | --- | --- | --- | --- | --- | --- | --- | --- | --- | --- | --- | --- | --- | --- | --- | --- | --- | --- | --- | --- | --- | --- | --- | --- | --- | --- | --- | --- | --- | --- | --- | --- | --- | --- | --- | --- | --- | --- | --- | --- | --- | --- | --- | --- | --- | --- | --- | --- | --- | --- | --- | --- | --- | --- | --- | --- | --- | --- | --- | --- | --- | --- | --- | --- | --- | --- | --- | --- | --- | --- | --- | --- | --- | --- | --- | --- | --- | --- | --- | --- | --- | --- | --- | --- | --- | --- | --- | --- |

Thank You
